# Using Digital Technologies to Facilitate Care Coordination Between Youth Mental Health Services: A Guide for Implementation

**DOI:** 10.3389/frhs.2021.745456

**Published:** 2021-11-18

**Authors:** Frank Iorfino, Sarah E. Piper, Ante Prodan, Haley M. LaMonica, Tracey A. Davenport, Grace Yeeun Lee, William Capon, Elizabeth M. Scott, Jo-An Occhipinti, Ian B. Hickie

**Affiliations:** ^1^Brain and Mind Centre, The University of Sydney, Sydney, NSW, Australia; ^2^Translational Health Research Institute, Western Sydney University, Sydney, NSW, Australia; ^3^School of Computer, Data and Mathematical Sciences, Western Sydney University, Sydney, NSW, Australia

**Keywords:** mental health, health services, digital technologies, health information technologies, health informatics, care coordination, systems science, implementation research

## Abstract

Enhanced care coordination is essential to improving access to and navigation between youth mental health services. By facilitating better communication and coordination within and between youth mental health services, the goal is to guide young people quickly to the level of care they need and reduce instances of those receiving inappropriate care (too much or too little), or no care at all. Yet, it is often unclear how this goal can be achieved in a scalable way in local regions. We recommend using technology-enabled care coordination to facilitate streamlined transitions for young people across primary, secondary, more specialised or hospital-based care. First, we describe how technology-enabled care coordination could be achieved through two fundamental shifts in current service provisions; a model of care which puts the person at the centre of their care; and a technology infrastructure that facilitates this model. Second, we detail how dynamic simulation modelling can be used to rapidly test the operational features of implementation and the likely impacts of technology-enabled care coordination in a local service environment. Combined with traditional implementation research, dynamic simulation modelling can facilitate the transformation of real-world services. This work demonstrates the benefits of creating a smart health service infrastructure with embedded dynamic simulation modelling to improve operational efficiency and clinical outcomes through participatory and data driven health service planning.

## Introduction

Globally, mental health systems are facing challenges which impact their capacity to deliver quality care (1–4). Long-standing challenges of poor access and coordination have been documented (3, 5), while more recent issues have emerged including inefficient and redundant practises, legacy software systems, and system inertia which hampers the adoption of innovation to enhance operational efficiency. Meanwhile, the demand for mental health care is continuing to rise, putting increased pressure on services and limiting their capacity to address many of these deep-seated problems and improve the quality-of-care people receive. The adoption and implementation of digital technologies to support the delivery of mental health care to reconcile these issues is being fast-tracked due to the COVID-19 pandemic (6–8). Thus, it is widely recognised that transformation across the mental health system is needed and that digital technologies will play a key role, however there is a lack of clarity about the way in which technologies could support the delivery of quality mental health care and how best to achieve it at a local level.

There is a fundamental mismatch between the nature of mental disorders among young people (i.e., dynamic and multi-faceted) and the way the mental health system operates to deliver care (i.e., rigid and siloed). Mental disorders are typically heterogeneous and the specific needs of young people are diverse, extending beyond specific illness syndromes (e.g., depression, anxiety) to include social and occupational functioning, suicidal thoughts and behaviours, substance misuse, and physical health (9). These multidimensional needs, particularly if left unaddressed, tend to complicate treatment, and contribute to the negative long-term impacts of these disorders (10). A young person will also follow a trajectory over time, which may oscillate between health and disorder as a function of complex vulnerability, protective, and treatment factors. Unfortunately, the youth mental health system is not set up in a way that recognises this dynamicity and complexity. Instead, most services and programs respond to discrete problems and are often poorly integrated. This creates a siloed and rigid system that delivers “episodic” rather than “continuous” care, leaving the young person to navigate the complexities of a disconnected health system alone (3, 11, 12). These difficulties leave young people at risk for receiving no care at all, or an inappropriate level of care whereby many tend to leave care having not fully recovered or not having their needs sufficiently addressed (13, 14).

This article aims to outline how technologies can be used to facilitate some critical transformations within the youth mental health system to improve care coordination. The overarching approach involves using technology-enabled care coordination so that local services across all the levels of care (e.g., primary, specialised) can be integrated in such a way as to ensure every young person who seeks help is able to easily and timely access the right level of care for their needs. Such transformations to existing health system infrastructures are disruptive and challenging, thus we detail the methodologies being used to do this in real world settings to serve as a blueprint for other local implementations.

## Technology-Enabled Care Coordination

There is a need to ensure that young people are accessing the appropriate level of care for their needs, and that this process is supported by a mental health system that is connected and coordinated. This implies the need for a mental health system that “wraps around” the individual through the integration of services, and a system that is strategically aligned to provide a clear, supported care pathway for consumers that places them at the centre of their own care (3), and provides continuous and streamlined transition as they travel across primary, specialist, and hospital-based care (depending on their illness pathway).

We argue that technology-enabled care coordination requires two fundamental shifts in current service provisions; (1) a model of care which puts the person at the centre of their care; and (2) a technology infrastructure that facilitates the model of care. A person-centred model of care shifts the focus from the service provider and traditional diagnostic classification systems to the consumer and their unique needs. An example of this model is the Brain and Mind Centre (BMC) Youth Model of Care which recognises that early onset mental ill health is a predictor of severe and recurrent mental disorders later in life (15), that comorbidity and subthreshold symptoms are common and must be accounted for in treatment approaches and aims to prevent progression to more advanced stages of illness (16–19). The BMC Youth Model utilises multidimensional assessment and treatment, a wider list of personalised treatment options, the promotion of consumer participation in their own mental health care (i.e., shared decision making), and the provision of earlier and more effective clinical interventions matched to the young person's needs (18–20). Importantly, the model promotes coordination between service providers by focusing on what the needs are for an individual and encouraging multidisciplinary team-based care for assessment and intervention. This moves away from service silos and towards care that is person-centred and focused on helping young people find the right type of care across a connected service system. The model aligns with the general paradigm shift in mental health towards, dimensional, transdiagnostic, and integrated care approaches which recognise the limitations of traditional nosology and seek to provide a models of psychopathology and care that more closely matches real-world illness trajectories and dynamics (21, 22). Other service models that adopt a similar philosophy to care provision will also be suitable for technology-enabled care coordination.

Digital health technologies (e.g., health-related internet-based platforms, apps, and e-tools) provide a promising way to address the practical barriers related to young people navigating the mental health care system, and can enable the practise of the BMC Youth Model through the improvement of the way young people are assessed, treated, and tracked in and across services (23, 24). While these technologies can be used to increase overall capacity by reducing human resources needed to provide some of the aspects of specialist mental health services (e.g., reducing time spent on administrative work by clinical staff to increase their capacity to see patients), they can also fundamentally shift the model of care to be more personalised and measurement-based. Research has shown that integrating into traditional services can greatly enhance the efficiency and quality of care provided (25). Further, by utilising technologies, services can more easily connect and communicate, ensuring that individuals receive the care that they require (e.g., across more than one service), and that they do not get “lost in the system.”

The InnoWell Platform is an example of an internet-based system that is designed to help individuals manage their health and well-being across the lifespan (26). It does this by collecting, tracking, and reporting health information back to the individual and to the health professional to encourage ongoing, collaborative care partnerships (27). All data collected by the Platform is made available to young people and their care service provider through the platform to support the young person's mental health and to promote collaborative care partnerships. Being transparent and clear about the ownership and privacy issues associated with this highly sensitive data is critical to ensure young people trust that their information is safe and being used appropriately (28). As we move further into the digital age of mental health care, it may be critical for young people to own and manage their own data (i.e., sharing and access rights) from a variety of sources to both maximise interoperability between service providers for seamless and responsive care pathways and protect privacy. Realising this type of digital future will require a multidisciplinary approach that involves health care professionals, service administrators, lawyers, and engineers to challenge existing technological, legal, and health system barriers.

Though we reference the InnoWell Platform as an exemplar digital health technology, other digital platforms may be suitable and fit for purpose if they are underpinned by similar clinical and scientific concepts for delivering highly personalised and measurement-based care (29). For this, digital technologies need to be used as a tool to break down the existing silos between services and improve the way services work together to provide more seamless and responsive pathways to care for young people (30). While increasing the capacity and resources for existing services will help, there are major gains that could be realised by using technologies to transform the way service operate (Table 1). These changes require a whole of system approach that challenges the traditional funding and health system structures to determine how we can leverage the accessibility, scalability, and standardisation of technologies so that services are more dynamic in the way they share information, manage resource allocation, and ultimately give young people more control and flexibility over how and when and from who they get care.

**Table 1:** Features of technology-enabled care coordination.

**Component**	**How does it enable the technology-enabled care coordination?**
Standardised and ongoing multidimensional online assessment	Provides consistent and comprehensive assessment of a young person's needs and prevents the need to “re-tell their story” to multiple service providers.
Reliable triage to facilitate personalised care pathways (matching level of care to the individual)	Efficient triage processes improve appointment attendance rates (31) and reduce the negative effect of wait times, including loss of motivation and lowered expectation of improving (32). Furthermore, accurate triage matches appropriate level of care to the individual's needs and manages demand by reserving more intense treatment for clients with greater need (33).
Personalised care plans for both individuals and health professional (identifying care team)	Care coordination initiatives (e.g., The Partners in Recovery model) utilises a “support facilitator” or technology capability to identify and coordinate a personalised treatment plan based on an individual's needs that may include multidisciplinary care providers. This allows services and the consumer to monitor response to treatment and guide shared decision-making. Such models of care reduce the rate of unmet needs (34).
Real-time data tracking	Health professionals that are notified of a clients' deterioration are more likely to intervene and improve outcomes by adjusting the type and intensity of treatment (35). For example, changes in functioning (measured by Social and Occupational Functioning Assessment Scale; SOFAS), symptom severity measured by the Kessler-10, or Quick Inventory of Depressive Symptomatology; QIDS), or suicide ideation (Suicidal Ideation Attributes Scale; SIDAS).
Video-visit functionality	Provides cost-effective assessment and monitoring of consumers that have poor access to in-person health care.
Shared and interoperable technology for assessment data and care plans	Electronic health records of patients can be securely and efficiently shared between various services to increase access of patient data and facilitate shared decision-making (36).

## A Way Forward—Technology-Enabled Care Coordination in Real-World Settings

Most mental health services recognise the need for transformation and innovation, however implementing such changes, particularly when they involve new digital technologies, is challenging. The introduction of new digital technologies requires a commitment of time in training consumers and health professionals in their use and integration into care structures which is often accompanied by an uncertainty regarding whether the required time and financial investment will deliver improvements in the quality, efficiency, and effectiveness of care. Additionally, health services are typically faced with complex funding structures, legacy technologies, and outdated health system structures that limit their flexibility, systemic insights, time horizon for operational decisions, and capacity for change. This reflects the slow pace of change and adoption of innovation observed across existing health systems.

**Figure 1:**
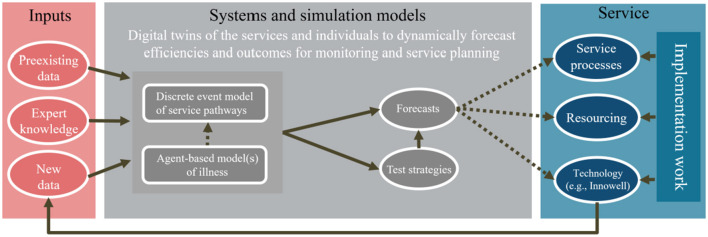
Establishing a sustainable monitoring framework that utilises DSM and health service implementation practises to iteratively improve service efficiency and performance.

We present a way forward which aims to bring together dynamic simulation modelling (DSM) with health service implementation research to create a smart health service infrastructure that can be used to guide service planning for optimal efficiency and improve pathways to care (Figure 1). This section outlines how this infrastructure has been applied to facilitate the implementation of technology-enabled care coordination. Local variation in context as a result of culture, policy, and funding will have a major influence on the way technology-enabled care coordination is implemented. Thus, we aim to demonstrate how through the combination of simulation modelling and participatory approaches, local service systems can come together to build partnerships, plan, and develop solutions that meet the needs of local stakeholders.

These methodologies have been employed within a group of local services representing a range of levels of care (e.g., primary, hospital, etc.), located in a similar geographical region (Sydney, Australia). These services provide an example of settings in which individuals would naturalistically be referred between for different mental health care needs. Participating services include: a nationally funded initiative (Primary Health Network-funded *headspace* centres); private specialist practice consortia (Mind Plasticity); private hospital provider (St Vincent's Private Hospital—USpace) and state-funded community-based specialist services (headspace early intervention team and early intervention in psychosis service).

### Dynamic Simulation Modelling

Dynamic simulation models (DSM) are computer models that are simplified representations of the real world. They have long been used in sectors such as engineering and economics, yet their use in mental health care is novel. DSM bring together a variety of sources of evidence, such as (i) *research evidence*; (ii) *expert and local knowledge*; (iii) *practise experience*; and, (iv) *primary and secondary data*, to map and quantify complex problems. Here, simulation allows us to test the likely impacts of technology-enabled care coordination within these services in conjunction with alternative service configurations, models of care, scheduling, and resource allocations before being implemented in the real world, to provide guidance for implementation and increase confidence in its potential value to the stakeholders involved. By reducing perceived complexity and integrating multiple diverse perspectives in unified systemic views of different granularity, simulation reduces cognitive load and improves efficacy in complex decision making.

For this work, an individual-centric approach to model design is used which combines discrete-event simulation and agent-based modelling. Discrete event simulation captures the workforce, resource, and operational aspects of the services, their processes and pathways with which individuals interact and receive care. Agent-based modelling is a computational approach whereby agents (i.e., young people, clinicians) are defined by a set of characteristics and rules that regulate how they behave and relate with each other. The global (system-level) behaviour then emerges as a result of the interaction of many agents with their environment (i.e., young people interacting with clinicians and services to receive care). These models allow us to consider heterogeneity (individual and environmental variation), feedback (where past experiences change the course of future responses), stochasticity (model unfolds in a probabilistic, as opposed to deterministic, fashion), resource constraints, and patient or information flows (37).

These models are developed using participatory design methodologies to collect qualitative data from various stakeholders regarding elements of health service pathways, processes, resources, and information flows. Stakeholders from participating services including young people, supportive others, health professionals, service managers, and service administrators inform the development of a conceptual map of the services and its elements. This conceptual map is used to create a computational model of these services (which exists as a “digital twin” of each service), whereby various model of care scenarios can be simulated (e.g., varying uses of digital technology, or varying workforce utilisation, etc), to explore likely impacts on key outcomes of interest such as recovery, disengagement from services, wait-times for interventions, etc. The information gathered from these participatory workshops inform the computational model structure and its parameters. The approach is ongoing and iterative whereby stakeholders are invited to interrogate the model architecture for critique and determine whether any further inputs (e.g., further information regarding client access, current workforce, typical referral pathways, etc). This process continues until a model structure is accepted by the service to guide service planning and implementation.

### Health Service Implementation and Evaluation

Implementing digital technologies is challenging and requires the participation of youth mental health services and their funding and administrative bodies. Therefore, intensive and ongoing health service implementation work is needed to facilitate the roll out of technology-enabled care coordination across a local service system to increase adoption and uptake.

As in the development of the DSM, a series of pre implementation workshops (service pathway and configuration) are conducted with participating services, including service administrators, health professionals, and individuals in their service population. This work focuses on ensuring that both components of the technology-enabled care coordination (model of care and technology) are embedded within services, to improve care coordination and continuity within and between the services. Service pathway workshops with each participating service (individually and collectively) are carried out to set up the technology (i.e., InnoWell Platform) to suit the participating service and address any changes required to service pathways between services. Service pathway workshops aim to collect data regarding the potential barriers and facilitators for each participating site when thinking about their implementation of the technology within their service pathway. These workshops characterise clinical pathways through services including intake, assessment, treatment planning and intervention, progress monitoring, and service movement/termination (38). These workshops should be informed by the insights generated by the DSM so that decisions about implementation and the likely workforce impacts are informed by the best available data and expert knowledge. Service and workforce impacts include (but are not limited to); new service pathways or processes (e.g., new referral procedures, use of technology for care planning meetings, changes to how care allocation occurs), reorganisation of staff resources (e.g., utilising senior clinical staff for triage and assessment or for brief interventions), or changes to the way data is collected so that less clinical time is spent on information gathering and more time is spent on interventions. Configuration workshops aim to determine the clinical content and set up of the technology to meet the needs of the local implementation (e.g., relevant clinical assessments, care options offered to individuals, and deciding on thresholds for notifying health professionals of suicidal thoughts and behaviour scores of individuals). This includes identifying the digital readiness and competence to use the technology at each service to determine the type and level education and training required.

The purpose of these pre-implementation workshops (both service pathway and configuration) is to inform the customisation and configuration of the technology, ready for implementation by participating services (39, 40). The outcome of this pre-implementation work will be a “future” service map for incorporating the technology to ensure smooth implementation and effective utilisation of the technology within the service pathway.

Once the technology has been implemented, quantitative and qualitative data are gathered to evaluate and monitor changes within participating services that are using the technology. These methods for evaluation and monitoring have been previously developed by researchers at The University of Sydney's Brain and Mind Centre, and utilised in other University of Sydney-sponsored research studies implementing the InnoWell Platform (41). The methods include: online surveys, semi-structured interviews, and group workshops with service staff, as well as implementation logs completed by the on-the-ground implementation officer. The online surveys, semi-structured interviews and group workshops collect data regarding the impact of the technology on the service, the social value of the technology, and the quality, usability and acceptability of the technology. The implementation logs collect data regarding service metrics (i.e., uptake and engagement), the capacity/readiness of staff, staff education and training, any adaptations that have been made or need to be made to the technology or service processes and pathways, barriers to adoption, and overall acceptance of the technology within the service.

## Conclusion

It's widely considered that the future of mental health care will include the use of digital technologies. Yet, careful consideration for how these technologies are implemented and used really matters if improvements to service efficiencies and clinical outcomes are to be achieved. The digital landscape is already a crowded space with different electronic medical record systems being used across a range of service providers, and new digital platforms and applications emerging rapidly. The danger is that digital technologies merely perpetuate the silos that already exist even further. We present the case for technology-enabled care coordination and illustrate how dynamic simulation modelling and health service implementation research is being used to facilitate its adoption and transformation of youth mental health services.

## Data Availability Statement

The original contributions presented in the study are included in the article/supplementary material, further inquiries can be directed to the corresponding author/s.

## Author Contributions

FI and SP wrote the manuscript. FI, SP, HL, TD, and GL developed the technology-enabled care coordination protocol. FI, AP, and J-AO designed the dynamic simulation model. ES and IH supervised the work and provided clinical and scientific leadership. WC designed and created the figure and table. All authors assisted with manuscript drafting and approved the final manuscript.

## Funding

This research was funded by the BUPA Health Foundation.

## Conflict of Interest

IH was an inaugural Commissioner on Australia's National Mental Health Commission (2012–18). He is the Co-Director, Health and Policy at the Brain and Mind Centre (BMC) University of Sydney. The BMC operates an early-intervention youth services at Camperdown under contract to headspace. He is the Chief Scientific Advisor to, and a 5% equity shareholder in, InnoWell Pty Ltd. InnoWell was formed by the University of Sydney (45% equity) and PwC (Australia; 45% equity) to deliver the $30 M Australian Government-funded Project Synergy (2017–20; a three-year program for the transformation of mental health services) and to lead transformation of mental health services internationally through the use of innovative technologies. ES was the Medical Director, Young Adult Mental Health Unit, St Vincent's Hospital Darlinghurst, Discipline Leader of Adult Mental Health, School of Medicine, University of Notre Dame, Principal Research Fellow, Brain and Mind Centre, The University of Sydney and Consultant Psychiatrist. She has received honoraria for educational seminars related to the clinical management of depressive disorders supported by Servier and Eli-Lilly pharmaceuticals. She has participated in a national advisory board for the antidepressant compound Pristiq, manufactured by Pfizer. She was the National Coordinator of an antidepressant trial sponsored by Servier. TD is now Director (Research and Evaluation), Design and Strategy Division, Australian Digital Health Agency. J-AO is Head of Systems Modelling, Simulation & Data Science, Brain and Mind Centre, University of Sydney, as well as Managing Director of Computer Simulation & Advanced Research Technologies (CSART). The remaining authors declare that the research was conducted in the absence of any commercial or financial relationships that could be construed as a potential conflict of interest.

## Publisher's Note

All claims expressed in this article are solely those of the authors and do not necessarily represent those of their affiliated organizations, or those of the publisher, the editors and the reviewers. Any product that may be evaluated in this article, or claim that may be made by its manufacturer, is not guaranteed or endorsed by the publisher.
